# Mitigating the impact of HIV/AIDS among in and out of school youth through peer education using family life HIV education in federal capital territory ABUJA NIGERIA

**DOI:** 10.1186/2047-2994-4-S1-P111

**Published:** 2015-06-16

**Authors:** FO Agbaje

**Affiliations:** 1 Programs, OROL Youth Empowerment Initiative, Lagos, Nigeria

## Introduction

HIV/AIDS has systematically permeated the entire Nigerian social fabric, affecting males and females in both urban and rural areas, as well as adolescent young people.

## Objectives

This paper presents findings from an evaluation of a HIV prevention program designed to determine the effects of HIV prevention intervention (HPI) on in school youths in AMAM Local Government Area Abuja Nigeria.

## Methods

Data were collected from 75 trained peer educators from three Schools in AMAC Local Government Area FCT Abuja Nigeria and also 100 in school youths who were not trained. Data were also collected at the beginning and end of the intervention among 950 in school youths that were reached with peer education in three Schools and also among other 1100 in school youths from three other schools that were not reached with the program using qualitative and quantitative research methods.

## Results

The findings revealed that the programme had several positive effects on the students such as increase in knowledge of HIV/AIDS, adoption of preventive behavior and acquisition of life skills. The quantitative data shows the knowledge of the respondents have increased by 71% at the end of the intervention and the result also shows that the students trained as peer educators have higher knowledge of HIV prevention and life skills than those who were not trained.

## Conclusion

The quantitative and qualitative data show that the project has produced several positive multiplier effects on the knowledge and behavior of youths. The findings also show It is necessary to focus on young people because they are at the center of the HIV/AIDS epidemic. The project shows that Peer education is one of the best approaches to providing comprehensive knowledge on HIV/AIDS/STIs and related issues, as it provide an excellent environment for effective peer-to-peer learning.

## Disclosure of interest

None declared.

